# Resilience, emotions, and suicidal ideation in Mexican adolescents during COVID-19 pandemic: risk factors and protective mechanisms

**DOI:** 10.3389/fpsyt.2024.1410873

**Published:** 2024-10-08

**Authors:** Ángel Alberto Puig-Lagunes, Guerson Yael Varela-Castillo, Juan Francisco Rodríguez-Landa, Fabiola Ortiz-Cruz, León Jesús German-Ponciano

**Affiliations:** ^1^ Facultad de Medicina, Universidad Veracruzana, Minatitlán, Mexico; ^2^ Programa de Doctorado en Neuroetología, Instituto de Neuroetología, Universidad Veracruzana, Xalapa, Mexico; ^3^ Laboratorio de Neurofarmacología, Instituto de Neuroetología, Universidad Veracruzana, Xalapa, Mexico; ^4^ Facultad de Odontología, Universidad Veracruzana, Minatitlán, Mexico; ^5^ Instituto de Neuroetología, Universidad Veracruzana, Xalapa, Mexico

**Keywords:** suicidal ideation, mental disorders, psychological resilience, adolescents, women, COVID-19

## Abstract

**Introduction:**

Suicide is the second leading cause of death among adolescents worldwide. Suicidal ideation (SI) in adolescents may be influenced by factors such as resilience, anxiety, and depressive symptoms. The COVID-19 pandemic served as a significant stressor with documented impacts, yet further research is needed to clarify its effects. While stressful events are known to be associated with increased SI, direct evidence linking COVID-19 specifically to elevated SI remains limited.

**Objective:**

The aim of this study was to identify the risk and protective factors associated with SI in Mexican adolescents during the COVID-19 pandemic.

**Method:**

A cross-sectional, correlational descriptive study was conducted between May and June 2022 with a sample of 2,194 high school students, with a mean age of 16.6 years (SD = 1.2). Among the participants, 58.9% were female and 41.1% were male. They completed inventories to assess stress, anxiety, depressive symptoms, and resilience.

**Results:**

Of 2194 adolescents, 15.5% presented SI, with females showing a higher prevalence of SI, anxiety, and depressive symptoms compared to males. In contrast, resilience was lower in females. Furthermore, depressive symptoms were identified as a risk factor for SI (OR 1.212; 95% CI 1.186, 1.240), whereas social competence was highlighted as a protective factor (OR 1.046 95% CI 1.044, 1.078).

**Conclusions:**

The findings underscore the importance of preventing SI in adolescents by addressing anxiety and depressive symptoms, which were identified as risk factors, and by promoting social competence as a protective factor. Therefore, an integrated approach that addresses both individual mental health and the social context must be considered for preventing SI in adolescents.

## 1 Introduction

According to the World Health Organization, suicide is the second leading cause of death among adolescents and young adults aged 15-29 years old (1). Suicidal ideation (SI) is a key predictor of suicide risk (2, 3) and is characterized by intrusive and repetitive thoughts about self-harm (4). During the COVID-19 pandemic, some studies have reported an increase in SI and related behaviors among adolescents (5, 6).

Pandemic-related stressors such as disrupted routines and social isolation have been linked to higher rates of adolescent SI. For example, Jo et al. (2023) found that lockdowns and school closures, which disrupted adolescents’ structured routines, were associated with increased SI rates in South Korea, the United States, Japan, and China (7). Similarly, a systematic review by Blázquez-Fernández et al. (2023) highlighted a correlation between heightened social isolation and elevated SI rates among adolescents across multiple countries (8). In Mexico, research has identified factors such as loneliness, being female, having divorced parents, substance use (tobacco and alcohol), and exposure to psychological and physical violence as contributing to a higher prevalence of SI during the pandemic (9). These findings are particularly concerning given that eighty percent of suicides occur in low- and middle-income countries like Mexico, where limited resources for early detection and treatment of SI, especially in rural and marginalized areas, hinder effective intervention. The disparities in suicide mortality across regions underscore the need for prevention policies tailored to local contexts (10). Addressing the rise in SI rates requires targeted interventions such as counseling services, crisis support lines, and school-based mental health programs to tackle the broader social and emotional challenges associated with SI.

Recent research has also indicated a significant increase in anxiety and depressive symptoms among adolescents due to the pandemic (11). Studies have shown a strong correlation between anxiety, depression, and suicidal behavior in this age group (12, 13). Adolescents experiencing anxiety often have heightened concerns about future situations, which can increase their vulnerability to SI or self-harm as a coping mechanism. Additionally, anxiety in adolescence frequently precedes or accompanies depression (14). This complex interplay between anxiety and depressive symptoms during the pandemic highlights the urgent need for comprehensive strategies that address these interconnected challenges. Effective interventions should not only address specific symptoms but also build resilience and overall psychological well-being, ensuring that adolescents are better equipped to manage stress and recover from adversity (15).

Resilience is the ability to adapt and maintain emotional and psychological stability after facing adverse situations (16). Rather than being a fixed trait, resilience is a dynamic process that involves reorganizing personal resources and overcoming challenges to sustain mental health despite prolonged or repeated adversity (17–19). Research has shown that higher resilience is associated with reduced SI and a lower prevalence of mental disorders (20). Conversely, impairments in resilience factors have been linked to an increased risk of SI and psychiatric disorders in countries such as the United States and China (21–25). Resilience encompasses various factors, including self-confidence, social competence, family and social support, and structure (23). Social competence, defined as an individual’s capacity to interact effectively with both peers and adults, encompasses key skills such as perspective-taking, social problem-solving, and emotion regulation. These abilities not only contribute to future societal functioning but also play a crucial role in fostering supportive networks and effective communication. By doing so, social competence helps mitigate the impact of adverse experiences and reduces the risk of mental illness (19, 23, 26, 27). However, the role of these resilience factors in SI among Mexican adolescents remains unexplored. Therefore, investigating these factors could provide insights into potential protective mechanisms against suicidal behavior, highlighting the need for further research in this population.

Considering the aforementioned factors, it becomes necessary to explore the relationship between SI, resilience, anxiety, and depressive symptomatology, as these aspects have received limited attention in the Mexican context. Consequently, the primary objective of this study was to identify the risk and protective factors associated with SI in Mexican adolescents during the COVID-19 pandemic. The revelation of such an association has the potential to facilitate the development of more effective preventive strategies against both SI and psychiatric disorders among adolescents.

## 2 Materials and methods

### 2.1 Design of the study and participants

A cross-sectional, observational, and correlational study was conducted among adolescents attending nine public high schools in southern Veracruz, Mexico, during May and June 2022. A convenience, non-probabilistic sampling method was employed, and students from the second to sixth semesters enrolled in the February to June 2022 term were included. Only those students who voluntarily agreed to participate and completed the inventories in their entirety were included in the study.

The selection of the timeframe for this study was driven by the necessity to document the residual impact of the pandemic on adolescents, who continued to experience notable psychological and social consequences. Despite the Mexican government’s adoption of an endemic approach as of April 2022, full recovery had not yet occurred, and presential classes remained limited. Furthermore, this interval offered logistical advantages for conducting the study and contacting educational institutions. Therefore, the selected period provided an updated and comprehensive perspective on the continued impact of the pandemic on adolescents, aiding in the understanding of risk and protective factors in this transitional phase. A total sample of 2,194 adolescents was obtained, of whom 1,293 (58.9%) were females and 901 (41.1%) males, with a mean age of 16.6 years.

### 2.2 Recruitment procedures

Following the establishment of a collaboration agreement between the high schools and the medical faculty, an invitation was extended to high school students to participate in the study. Only students who had obtained informed consent from their parents and who had indicated their assent by the appropriate boxes, one for the parent and one for the student, were eligible to complete the questionnaire in Google Forms. Those who refused to respond or not completed questionnaires were excluded from the study. The questionnaire encompassed assessments of anxiety and depressive and stress symptoms, as well as resilience. Participants were informed about the research’s significance and encouraged to respond with honesty and sincerity. Additionally, we assured them of data confidentiality in compliance with the Federal Law for the Protection of Personal Data in Possession of Individuals, as approved by the National Chamber of Deputies (28).

### 2.3 Ethics

This research was approved by the Research Bioethics Committee of the Faculty of Medicine of the Universidad Veracruzana, Minatitlán campus (F-001-CI-2022). Furthermore, the research was conducted in accordance with the General Health Law of Mexico, specifically articles 13, 14, 16, 20, and 36, as well as the ethical standards set forth in the Declaration of Helsinki and the General Health Law of Mexico, particularly chapters 96, 100, and 102 of the research (29, 30).

### 2.4 Instruments

A questionnaire was constructed to elicit information pertaining to sociodemographic characteristics, including age, gender, academic semester, family composition, household size, physical activity, hobbies, employment status, and the educational level of the household head.

Moreover, the Patient Health Questionnaire-9 (PHQ-9) was selected because it is one of the most widely used clinimetric tools worldwide and Mexican population for the diagnosis and determination of the severity of depressive symptoms as well as the presence of SI (31). Question 9 of the PHQ-9 was employed to ascertain SI (32, 33). The questionnaire also inquired as to whether, over the previous two weeks, respondents had contemplated their own mortality. This variable was analyzed according to two categories: 1) No, which included responses indicating “Never,” and 2) Yes, which encompassed instances where students reported experiencing suicidal or death ideation on some days, more than half of the days, or almost every day within the last two weeks. The PHQ-9 has been shown to have high internal consistency. Cronbach’s alpha ranges from 0.86 to 0.89 (31–33).

The Depression, Anxiety, and Stress Scale-21 (DASS-21) is one of the most widely used scales worldwide in clinical and non-clinical settings. It is well suited for research purposes and has adequate psychometric values (34). It has been used to assess the presence and severity of depressive, anxiety, and stress-related symptoms (35). The scale consists of 21 items, including three subscales of seven items, scored from 0 to 3. Scores for each subscale are summed and interpreted separately. In addition, the cutoff scores proposed by Román et al. (2016) (36) were used to identify adolescents at risk for mental health problems. The DASS-21 has shown excellent reliability, with a Cronbach’s alpha of 0.93 for the depression subscale, 0.90 for anxiety, and 0.92 for stress (34–36).

Similarly, the Mexican Resilience Scale (RESI-M) is a scale widely used in research and is one of two scales: the Connor-Davidson Resilience Scale and the Resilience Scale for Adults (36). This adaptation used, consisting of 43 items organized into five factors: 1. Strength and self-confidence, 2. Social competence, 3. Family support, 4. Social support, and 5. Structure. The scale has a Cronbach’s alpha total consistency of 0.93, which explains 43.60% of the variance (23).

### 2.5 Statistical analyses

Descriptive statistics were used to analyze the results, which are reported as frequencies and percentages. In addition, two binomial logistic regression models (BRLM) were fitted and estimated to determine which of the variables evaluated predicted SI. The response variable in the model was SI, which is dichotomous (presence or absence) and was predicted based on sociodemographic factors, resilience, anxiety and depressive symptoms variables.

The results indicated whether depressive symptoms and gender can increase the probability of developing SI; and, conversely, whether the presence of any resilience factor predicts not developing SI. For these models, an analog of Nagelkerke’s R2 was calculated. Additionally, model assumptions were evaluated (linearity, independence of errors, and absence of multicollinearity). The significance criterion was an α p ≤ 0.05. The data were analyzed using the R Studio Version 1.2.1335 statistical package.

## 3 Results

Of the total sample (n=2,194), 703 (32%) adolescents were enrolled in the second semester, 619 (28.2%) in the fourth semester, and 872 (39.7%) in the sixth semester of high school. Furthermore, 305 adolescents (13.9%) indicated that, in addition to their academic studies, they were engaged in employment. Most of these adolescents came from a nuclear family structure (63.8%) (**Table 1**).

**Table 1:** Sociodemographic factors of adolescents included in the study from southern Veracruz during the COVID-19 pandemic.

		n	%
**Occupation**	Student only	1889	86.1
	Studying and working	305	13.9
**Family composition**	Single parent	793	36.1
	Nuclear	1401	63.8
**Home inhabitants**	2	113	5.1
	3	424	19.3
	4	789	36.0
	5 or more	868	39.6
**Exercise**	No	832	37.9
	Yes	1362	62.1
**Hobbies**	No	377	17.2
	Yes	1817	82.8

The DASS-21 results revealed that 38.5% of adolescents exhibited symptoms of anxiety, while 23.9% experienced some degree of stress. Additionally, 33.7% of the participants presented depressive symptoms (**Table 2**).

**Table 2:** Anxiety, depression, and stress symptoms in high school adolescents studied during the COVID-19 pandemic.

Symptoms/ Category	None/Mild	Moderate	Severe	Extremely Severe
	n (%)	n (%)	n (%)	n (%)
**Anxiety**	1350 (61.5%)	277 (12.6%)	149 (6.8%)	418 (19.1%)
**Depression**	1454 (66.3%)	289 (13.2%)	183 (8.3%)	268 (12.2%)
**Stress**	1669 (76.1%)	217 (9.9%)	250 (11.4%)	58 (2.6%)

In the study population, the structure factor was most found at high levels, indicating that a high percentage of adolescents demonstrated strong stability and organization in their lives. In contrast, the family support factor was most frequently observed at low levels, with students experiencing limited family support (**Table 3**).

**Table 3:** Distribution of resilience levels and its factors in adolescents studied during COVID-19 pandemic.

RESI-M Factors	Low	Medium	High
	n (%)	n (%)	n (%)
**Strength and self-confidence**	179 (8.2%)	969 (44.2%)	1046 (47.7%)
**Social competence**	198 (9.0%)	938 (42.8%)	1058 (48.2%)
**Family support**	385 (17.5%)	1014 (46.2%)	795 (36.2%)
**Social support**	247 (11.3%)	910 (41.5%)	1037 (47.3%)
**Structure**	367 (16.7%)	1184 (54.0%)	643 (29.3%)
**Resilience**	119 (5.4%)	1067 (48.6%)	1008 (45.9%)

Regarding SI, it was observed that 340 (15.5%) adolescents indicated having considered suicide during the last few weeks. This was predominantly in females (18.1%) compared to males (11.6%; X2 = 17.24, p <.001). However, no associations were identified between other sociodemographic variables and the development of SI. A positive association between SI and anxiety, depressive symptoms, and stress was observed in women (p <.001). Conversely, adolescents with higher resilience levels exhibited lower SI (**Table 4**).

**Table 4:** Gender distribution of SI, anxiety, depression, and stress symptoms, and resilience in adolescents studied during the COVID-19 pandemic.

	Men (n=901)		Women (n=1293)		
	With SI 105 (11.6%)	Without SI 796 (88.4%)	With SI 235 (18.1%)	Without SI 1058 (81.9%)	x^2^; p -Value
Variables	n (%)	n (%)	n (%)	n (%)	
**Anxiety**	63 (60%)	199 (25%)	176 (74.79%)	406 (38.37%)	x^2^ = 67.82; p <.001*
**Depression**	69 (65.71%)	173 (21.73%)	182 (77.44%)	316 (29.68%)	x^2^ = 39.02; p <.001*
**Stress**	32 (30.47%)	103 (12.93%)	144 (61.27%)	239 (22.58%)	x^2^ = 65.87; p <.001*
**Strength and self-confidence**	35 (33.33%)	434 (54.52%)	51 (21.70%)	526 (49.71%)	x^2^ = 14.82; p <.001*
**Social competence**	39 (37.14%)	473 (59.4%)	76 (32.34%)	470 (44.42%)	x^2^ = 45.58; p <.001*
**Family support**	18 (17.14%)	344 (43.21%)	33 (14.04%)	400 (37.80%)	x^2^ = 10.44; p <.005*
**Social support**	38 (36.19%)	403 (50.50%)	62 (26.38%)	534 (50.47%)	x^2^ = 1.76; p = .414
**Structure**	32 (30.47%)	294 (36.93%)	21 (8.93%)	296 (27.97%)	x^2^ = 23.78; p <.001*
**Resilience**	27 (25.71%)	434 (54.52%)	44 (18.72%)	503 (47.54%)	x^2^ = 17.35; p <.001*

The values shown in the anxiety, stress, and depression symptoms correspond to moderate, severe, and extreme levels; on the other hand, the resilience factors represent the adolescents who presented high levels. *Indicates a significance value of p < .001 or p < .05.

### 3.1 Logistic regression models for SI

The initial regression model employed the DASS-21 factors as predictors, yielding a variance of 30%. This analysis identified depressive symptoms (p <.001) as a risk factor for the development of SI (OR 1.212; 95% CI 1.186, 1.240), indicating individuals experiencing depressive symptoms exhibited a 54% increased probability of developing SI. Furthermore, the data indicated that being a woman (p <.05) was associated with an increased probability of developing SI by.56 (OR 1.278; 95% CI 0.974, 1.683).

The second model considered the RESI-M factors as protective variables, which explained 19% of the variance. The results indicated that strength and self-confidence, social competence, and family support were significant predictors of the absence of SI development, in comparison to other resilience scale dimensions and variables evaluated in the study (p <.05). Nevertheless, social competence (OR 1.046 95% CI 1.044, 1.078) was the sole factor that, in addition to the aforementioned variables, served to protect against the development of SI with a probability of.51 (**Table 5**).

**Table 5:** Logistic regression model with protective factors for the development of SI.

Predictor	OR	CI 95%	p-value
**Family support**	.845	[.815,.876]	p <.001*
**Social competence**	1.046	[1.044, 1.078]	p <.001*
**Strength and self-confidence**	.963	[.888,.949]	p <.001*
Risk factor	OR	CI 95%	p-value
**Depression**	1.212	[1.186, 1.240]	p <.001*
**Gender (Women)**	1.278	[0.974, 1.683]	p <.05*

*Indicates a significance value of p < .001 or p < .05.

## 4 Discussion

During the COVID-19 pandemic, research has shown an increase in SI and related behaviors among adolescents (5, 6). However, in Mexico, the relationship between anxiety, and depressive symptoms as well as SI has not been thoroughly explored, nor have coping strategies such as resilience. Due to this, our study identified the risk and protective factors associated with SI in Mexican adolescents during this period. The results provide key insights to develop more effective preventive strategies for SI in this population.

The prevalence of SI among adolescents in this study surpasses the 14% reported in a study involving adolescents from 82 countries during the first year of the COVID-19 pandemic (37) and exceeds the 7.3% and 14.9% prevalence rates documented in Mexico in 2018 and 2021, respectively. This increase appears linked to the ongoing pandemic’s impact on adolescents’ mental health (9, 38). The correlation between the pandemic and the rise in SI underscores the importance of external factors in shaping the psychological well-being of this demographic. Given this context, understanding how variables such as mental illness symptoms or protective factors like resilience and social support interact is crucial for designing effective interventions. These interventions should not only address the immediate effects of the pandemic but also strengthen adolescents’ psychological and social resources to mitigate the negative impacts of future crises.

Depressive symptoms in our study were significantly higher than that reported in studies conducted in Mexico in 2018 and 2021, which reported rates of 13.3% and 13.4%, respectively (9, 38). The discrepancy may be attributed to the disparate measurement tools employed, with previous studies utilizing the CES-D and our study employing the DASS-21. While these scales categorize depressive symptoms in disparate ways, which could account for some discrepancy (39), studies like Wilson et al. (2014) have found comparable prevalence rates when both scales are applied to the same sample, indicating their comparability in certain contexts (40). Furthermore, Neyazi et al. (2024) identified robust convergent validity between the DASS-21 and CES-D, confirming that both tools effectively assess depressive symptoms in a similar manner (41). These findings underscore the necessity of understanding how these measurement tools, in conjunction with contextual factors, may influence the interpretation of results. This highlights the importance of a comprehensive approach to the assessment of adolescent mental health. However, these results could also be explained by temporal factors, such as the onset and prolongation of the pandemic, which created a particularly challenging environment for adolescents. The prolonged exposure to health measures such as confinement, isolation, quarantine, the lack of recreational activities, and other stressors over time, including uncertainty about infection, loss of family members, or economic instability, likely contributed to the observed increase in depressive symptoms (42). These results imply that the cumulative impact of pandemic-related stressors necessitates targeted mental health interventions for adolescents. Addressing both immediate and long-term psychological effects, including the implementation of support systems to manage depressive symptoms, will be essential in mitigating the impact of similar future crises.

Additionally, our results revealed that women are more likely than men to develop SI, consistent with previous research on adolescents in Mexico (9). This discrepancy is linked to various factors, including women’s tendency to internalize emotional distress. Early pubertal development of sexual characteristics in females can lead to experiences of being scrutinized, often resulting in maladaptive coping strategies such as rumination and difficulties in understanding their own emotions (26). Another factor contributing to the higher prevalence of SI among women is their greater exposure to stressful circumstances and maltreatment (9). In Mexico, 70.1% of women experience gender-based violence, including physical, psychological, sexual, and economic abuse (43). Patriarchal norms and economic inequality worsen this violence and restrict access to support. Although men also face abuse, women encounter a broader and more severe range, with greater barriers to justice and protection. Another factor associated with the higher prevalence of SI in women compared to men is hormonal fluctuations (26). Specifically, research has shown that SI in women is linked to the beginning of the follicular phase during menstruation, when levels of gonadal hormones, such as progesterone, are low (44). Although these results highlight a higher prevalence of SI in women compared to men, most studies have examined factors at an international level, with limited focus on the Mexican context. Investigating these factors within Mexico would enhance the understanding of this prevalence and enable more effective adjustment of intervention strategies for preventing SI in Mexican adolescents.

As with other reports, the data indicate that anxiety symptoms are associated with an elevated risk of SI in this population (26). Additionally, depressive symptoms have been identified as the most robust predictor of SI in Mexican adolescents (9, 13). These findings corroborate those of previous studies and underscore the necessity of addressing these psychiatric disorders to mitigate SI. However, it is fundamental to investigate the role of specific factors, such as family cohesion, traumatic events, and social support networks, in influencing these associations in Mexican adolescents. Collectively, these results provide a robust foundation for future research and the development of efficacious support and prevention programs.

In addition to examining the risk factors associated with the prevalence of SI in adolescents, exploring the protective factors that can reduce these tendencies is necessary. In this context, the concept of resilience plays a pivotal role. Our study not only revealed that the adolescents with higher levels of resilience had lower SI but also highlighted a specific facet of resilience that stood out among the adolescents under investigation – social competence. This observation aligns with the findings of other studies that have demonstrated a negative correlation between the number of social skills and the likelihood of suicide risk in adolescents (45, 46). It is noteworthy that this is the first study in Mexico to report the impact of social skills and their association with a lower prevalence of SI in adolescents. This underscores the necessity to design and implement intervention programs centered on the enhancement of socioemotional abilities. For example, prior studies in various countries have demonstrated the efficacy of intervention programs in reducing anxiety and depressive symptoms among adolescents (27, 47), suggesting that such programs could serve as pivotal strategies for preventing SI. In line with this, Nunes & Mota (2023) emphasized the importance of social competence as a protective factor against SI (48). Their study found that skills such as assertiveness and empathy not only help adolescents manage emotional distress but also moderate the relationship between parenting styles and suicidal thoughts. Specifically, while maternal physical coercion was positively correlated with SI, assertiveness showed a negative association with these thoughts. Moreover, empathy emerged as a crucial moderator in the relationship between both authoritative and authoritarian parenting styles and SI, highlighting the potential for targeted programs to strengthen social skills as part of comprehensive prevention efforts. This underscores the value of integrating emotional and social competence training into broader mental health interventions to effectively reduce the risk of SI in adolescents.

Suicidal behavior may arise from a complex interplay of biological, psychological, and sociocultural factors experienced by an individual with a unique history and specific circumstances that evolve over time (27, 46). Other resilience factors, such as family support (49) and self-confidence (24), which have been linked to protective effects against SI (50), may not have been significant in this study.

## 5 Limitations and future lines of research

The limitations of this study should be acknowledged. Firstly, the research was conducted exclusively with students from public schools, which highlights the necessity to include diverse populations and educational levels in future studies for a more comprehensive understanding. Secondly, the cross-sectional design provides a snapshot at a single point in time, which limits the ability to observe changes over time. Furthermore, the use of online questionnaires may have affected the representativeness of the sample, as only adolescents with internet access and familiarity with technology could participate. Finally, while associations were identified between SI, anxiety, and depressive symptoms as well as social competence, these relationships should not be interpreted as causal and may be influenced by other unmeasured factors.

## 6 Conclusion

Our findings underscore the necessity for a comprehensive approach to address SI among adolescents in Mexico. The high prevalence of SI, along with elevated levels of anxiety and depressive symptoms and their positive correlation, highlights the necessity for the development and implementation of targeted intervention strategies. These strategies should address not only anxiety and depressive symptoms but also the promotion of socio-emotional skills. Social competence, identified as a protective factor, should be a central component of support and prevention programs. Furthermore, the increased vulnerability of females related to SI emphasizes the importance of designing interventions that account for gender differences and address the specific needs of this group. The implementation of these strategies could help reduce the prevalence of SI and improve the overall well-being of adolescents.

## Data Availability

The raw data supporting the conclusions of this article will be made available by the authors, without undue reservation.
